# Algorithm-supported visual error correction (AVEC) of heart rate measurements in dogs, *Canis lupus familiaris*

**DOI:** 10.3758/s13428-014-0546-z

**Published:** 2014-12-25

**Authors:** Iris Schöberl, Kim Kortekaas, Franz F. Schöberl, Kurt Kotrschal

**Affiliations:** 1 Department of Behavioral Biology, University of Vienna, Althanstrasse 14, 1090 Vienna, Austria; 2 Wolf Science Center, Ernstbrunn, Austria; 3 Faculty of Physics, University of Vienna, Vienna, Austria; 4 Konrad Lorenz Research Station, Grünau im Almtal, Austria

**Keywords:** Domestic dog, Heart rate, Error correction

## Abstract

Dog heart rate (HR) is characterized by a respiratory sinus arrhythmia, and therefore makes an automatic algorithm for error correction of HR measurements hard to apply. Here, we present a new method of error correction for HR data collected with the Polar system, including (1) visual inspection of the data, (2) a standardized way to decide with the aid of an algorithm whether or not a value is an outlier (i.e., “error”), and (3) the subsequent removal of this error from the data set. We applied our new error correction method to the HR data of 24 dogs and compared the uncorrected and corrected data, as well as the algorithm-supported visual error correction (AVEC) with the Polar error correction. The results showed that fewer values were identified as errors after AVEC than after the Polar error correction (*p* < .001). After AVEC, the HR standard deviation and variability (HRV; i.e., RMSSD, pNN50, and SDNN) were significantly greater than after correction by the Polar tool (all *p* < .001). Furthermore, the HR data strings with deleted values seemed to be closer to the original data than were those with inserted means. We concluded that our method of error correction is more suitable for dog HR and HR variability than is the customized Polar error correction, especially because AVEC decreases the likelihood of Type I errors, preserves the natural variability in HR, and does not lead to a time shift in the data.

Heart rate (HR) and heart rate variability (HRV) are frequently used to measure stress responses in humans and nonhuman animals. The sympathetic activity is reflected by HR, with a high HR indicating high arousal. In contrast, HRV gives information about autonomic flexibility and reflects the capacity for regulated emotional responding (Appelhans & Luecken, 2006); hence, HRV is considered an indicator of welfare status in animals (von Borell et al., 2007). A common method, particularly in medical diagnosis, is to measure HR by electrocardiography (ECG). However, such systems are hard to apply in unrestrained situations, especially with animals. As an alternative, chest-belt-based systems designed for monitoring HR and HRV in exercising humans are increasingly used. In fact, the Polar HR monitor RS800CX has been validated for dogs, and its results are generally comparable with conventional ECG data (Essner, Sjöström, Ahlgren, & Lindmark, 2013; Jonckheer-Sheehy, Vinke, & Ortolani, 2012). In pigs, it has been shown that HR measures from the Polar were only as reliable as ECG data when they were carefully corrected for outliers (Marchant-Forde, Marlin, & Marchant-Forde, 2004).

An outlier is defined by its deviating position relative to the distribution of the data in a set and to the general variability of the data (Barnett & Lewis, 1994; Davies & Gather, 1993; Schendera, 2007). Outliers may either be part of the biological variability of the parameter measured or be due to measurement error. In the case of HR, a natural outlier can be caused by stress-induced arrhythmia, based on a disruption of the regulated electrical activity in the heart. Outliers due to measurement errors may be caused by the bodily movement of animals, leading to unstable signal transmission between the body surface and the electrodes in the belt, or by electromagnetic fields of various sources (Marchant-Forde et al., 2004). These errors can significantly influence analyses of HR frequency, for the time domain in particular (Berntson & Stowell, 1998).

The effects of errors on the quality of HR parameters are greater, the more frequent the errors are and the shorter the measurement period is (Marchant-Forde et al., 2004). Occasional outliers due to measurement errors may not affect the mean HR too much, but they will still strongly affect the statistical results, particularly when using parametric tests (Schendera, 2007). Also, outliers will have a large effect on HRV and spectral analysis (Berntson & Stowell, 1998). Finally, even minor errors, which might not influence the statistical parameters themselves, may influence the goodness of fit of a statistical model, and thus affect the choice of model (Buttler, 1996).

Some errors are easily identified by the naked eye because they fall outside of their expected range and are discontinuous with the neighboring values. High standard deviations may hint at outliers, too (Schendera, 2007). However, most of the measurement errors may be relatively inconspicuous and may need to be detected as deviations from their surrounding values and trends. If outliers exceed a certain frequency, any mathematical algorithm calculation will not easily be able to identify them as errors (Berntson, Quigley, Jang, & Boysen, 1990), pointing to the importance of initial visual inspection of the graphical representation of any data set. Checking for outliers also means judging the plausibility of the entire data set, which should be done by an expert who is familiar with the particular kind of data and the methods used to collect them (Schendera, 2007).

When deciding how to proceed with such “expert-identified” errors, the kind of data collected and the information to be retrieved have to be taken into consideration. For example, the decision to replace an error with a mean value depends on the type of the error (Marchant-Forde et al., 2004). In general, data files with more than 5 % errors should be excluded (Mulder, 1992). This suggests that a careful approach to error correction is particularly advisable in dogs, among which even different breeds may differ in HRV (Doxey & Boswood, 2004). Furthermore, the HR of adult dogs varies frequently in the form of a sinus arrhythmia, due to the influence of breathing on HR (Doxey & Boswood, 2004; Hamlin, Roger Smith, & Smetzer, 1966; Hanton & Rabemampianina, 2006; Shykoff, Naqvi, Menon, & Slutsky, 1991), which makes an automatic algorithm for error correction hard to apply. Unfortunately, in most studies on HR in dogs, no information is given about whether or how error correction was done (Beerda, Schilder, van Hooff, de Vries, & Mol, 1998; Gerth, Redman, Speakman, Jackson, & Starck, 2010; Handlin et al., 2011; Maros, Dóka, & Miklósi, 2008; Palestrini, Prato Previde, Spiezio, & Verga, 2005; Rehn & Keeling, 2011; Vincent & Leahy, 1997). The only study where this information was given (Kuhne, Hossler, & Struwe, 2014), the Polar software was employed for error correction.

The Polar system offers an automatic error correction tool in the “Polar ProTrainer 5 program.” However, this automatic error correction system was developed for human HR data. It is questionable whether this system is appropriate to correct dog HR data, as well. To date, no alternative correction method has been available for dog HR measured with the Polar system. For the reasons discussed above, the type of error correction chosen has a great impact on further analysis. In fact, the appropriateness of a method for finding outliers strongly depends on the data set (Barnett & Lewis, 1994; Schendera, 2007). Here, we propose a new and optimized method for error correction of HR and HRV in dogs.

## Methodology

### Ethical approval

Participation in our study was voluntary; dog owners were informed that they could stop the test situation at any time and were also asked to sign an information and consent form. Data collection was conducted according to the standards of the *Guide for the Care and Use of Laboratory Animals* published by the US National Institutes of Health (NIH publication no. 83-23, revised 1996) and of the German Society of Psychology (Ethische Richtlinien der DGPs und des BDP). Ethical review was done by the animal-welfare committee of the Faculty of Life Sciences, University of Vienna (approval number: 2014-015).

### Subjects and general procedure

Twenty-four owner–dog dyads participated in two separate meetings. HR and HRV data from intact male pet dogs (mean age ± *SD*: 3.64 ± 1.28 years; mean weight ± *SD*: 34.25 ± 15.05 kg), measured during the second meeting, were used to investigate the effects of error correction procedures.

### Data collection

The Polar HR monitor RS800CX was used to measure dog HR and HRV. This monitor records values from 15 to 240 beats per minute (bpm) on a beat-to-beat basis, with an accuracy of ±1 ms. The Polar system includes a chest belt with electrodes to measure HR. The information is then transmitted via a wireless connection to a data-logger watch, where the data are saved and transferred to a computer for permanent storage and analysis.

The owner applied the HR monitor belt to the dog’s chest and additionally fixed it with a standard dog harness. The Polar belt was worn for about 1 h. To improve signal transduction through the fur, an ultrasound gel (Henry Schein) was used to wet the skin. HR and HRV were first measured during 5 min of owner–dog play and 5 min of rest, which served as an adaptation period to the Polar belt. Then the dog experienced two staged threat situations in a counterbalanced order, one with and one without the owner present. After the second threat, the dog was pacified by the previously threatening person by talking to it in a friendly manner and offering cheese. Every test situation was followed by a recovery period of 15 min, during which the dog could move around freely. Error correction trials for HR and HRV were carried out on the data from the threat situations and the following 5 min of the recovery period.

### Error correction procedure

**Fig. 1:**
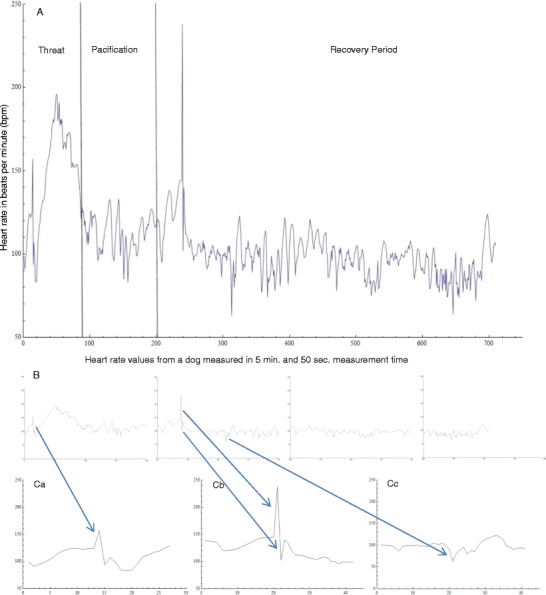
Graphical representation of heart rate (HR) values for visual inspection of a data set. (**A**) Dog HR from one complete threat situation, including the pacification and 5-min recovery periods. (**B**) Parts of Graph A, divided into blocks of 200 data points. (**C**) **a.** Value 14 (157 bpm) was identified as an error by the algorithm. **b.** Values 239 (238 bpm) and 240 (102 bpm) were deleted as errors after visual inspection, because of their clear discontinuity with the preceding and following values. **c.** Value 313 (63 bpm) was identified as an error by the algorithm

The HR data, in bpm and milliseconds between beats, were exported from the Polar Pro Trainer 5 program into Excel for further analysis. HR data was then imported into Mathematica 9. Afterward the data were visually inspected. To do so, a string of 200 values was visualized in an *x*/*y* plot (Fig. 1), and errors that could be clearly discriminated by eye (sharp peaks with extreme values as compared to the rest of the data file) were directly deleted in Excel (Fig. 1Cb). In unclear cases, we used the mathematical algorithm of Graf and Henning (1958, p. 8), as applied in Hultzsch (1966, p. 52):

$$ \left|{x}_g-\overline{x}\right|>k\left|s\right|. $$

*x*_*g*_ is an outlier, if the above equation holds, with *s* as the standard deviation, $\overline{x}$ as the mean value (excluding *x*_*g*_), and *k* given by (see Graf & Henning, 1958, p. 8):

$$ k=\frac{2\left(n-1\right)}{2\left(n-1\right)-{\lambda}_b^2}\left[{\lambda}_a+{\lambda}_b\sqrt{\frac{n{\lambda}_a^2+2\left(n-1\right)-{\lambda}_b^2}{2n\left(n-1\right)}}\right], $$

with *n* as the number of values, and *λ*_*a*_, *λ*_*b*_ are the solutions of the equations
where the error function (also called the *Gauss error function*) *Erf* ($\frac{\lambda }{\sqrt{2}}$) is defined by (Abramowitz & Stegun, 1970, p. 297)

$$ Erf\left(\frac{\lambda }{\sqrt{2}}\right)=\frac{1}{\sqrt{2\pi }}{\displaystyle {\int}_{-\lambda}^{+\lambda }{e}^{-{t}^2/2}dt}, $$

and *P* is the confidence level. A confidence level *P* has to be assumed for this calculation. The decision of which value to take for *P* is not a question of statistics, but an economic/biological question that should fit the goal of the error correction (Hultzsch, 1966). Depending on the data, a confidence level should be chosen that is appropriate to identify an error. With a higher *P*, the probability of including a value that is an outlier is higher, and with a lower *P*, the probability of excluding a value that is not an outlier is higher. Hence, the lower the *P*, the more conservative the analysis will be. With a 75 % confidence level, we chose a relatively conservative approach, because a missed outlier may have a greater influence on the data set than a deleted value that is not actually an outlier (Berntson et al., 1990).

We used the preceding and subsequent 20 values around a potential outlier for the error correction. We chose such a short period because of the generally high variability of dog HR. Furthermore, this string of 41 values approximates a 30-s measurement period, which was equivalent to the duration of the threat situation. We used a single-step procedure, which meant that all outliers relevant for the test period were identified in a single step instead of through successive identification and elimination (Davies & Gather, 1993). If a value was identified as an error by our algorithm, it was manually deleted. In cases in which the string of 41 values was expected to contain more than one error, the most extreme value was corrected first. In cases that were unclear even after application of the algorithm, the researcher, and not the program, ultimately decided whether or not to label a value as an error. The biological reasoning should be more relevant than mathematical algorithms, which cannot account for natural variations in dog HR; for instance, if a pattern exists within the data, which fits to the biological range of dog HR, one could decide not to correct the values, even if they were outliers according to the algorithm. In cases in which consecutive errors had to be deleted, the split data set was combined again before further analysis. Following a longstanding rule, files with more than 5 % errors were generally excluded from further analysis (Mulder, 1992).

### Data analysis

We conducted an exploratory analysis of HR error correction for 24 dogs, and also statistical comparisons between the Polar error correction and the AVEC. The percentages of errors were calculated, as well as the mean, median, and standard deviation of HR before and after both error correction methods. Similarly, HRV parameters, such as the SDNN, RMSSD, and pNN50 (for detailed information on HRV parameters and their definitions, see Malik, 1996), were calculated with Kubios HRV Version 2 (2008) before and after both error correction methods. Subsequently, the parameters were compared before and after AVEC and before and after the Polar error correction. Furthermore, the AVEC results were compared with those from the Polar error correction. For the Polar error correction, we used the initialized error correction with “Filter Power: Moderate” and “Minimum Protection Zone 6” of the Polar Pro Trainer 5 program. In addition, the data were compared after deleting errors and after replacing them by a mean. To do so, we used the data of one dog for which no errors were identified at all, and either randomly deleted 1 %, 2 %, 3 %, 4 %, and 5 % of the values or replaced them with a mean value calculated from the preceding and subsequent five values.

## Results

### Comparison of error correction methods

When using our AVEC method for the two test situations, the percentages of errors differed neither between the threats with and without the owner present (Wilcoxon signed-rank test: *n* = 24, *Z* = –1.086, *p* = .278) nor between the orders of testing (Wilcoxon signed-rank test: *n* = 24, *Z* = –0.629, *p* = .530) in the 24 dogs analyzed. In Threat Situation 1, the HR data of one dog included more than 5 % errors; the HR data of three dogs had more than 3 % errors. For Threat 2, the HR data of one dog had more than 3 % errors after the AVEC method. Since one of the dogs from Threat 1 had to be excluded from further analysis, only the results from the second threat will be used for further comparisons.

**Fig. 2:**
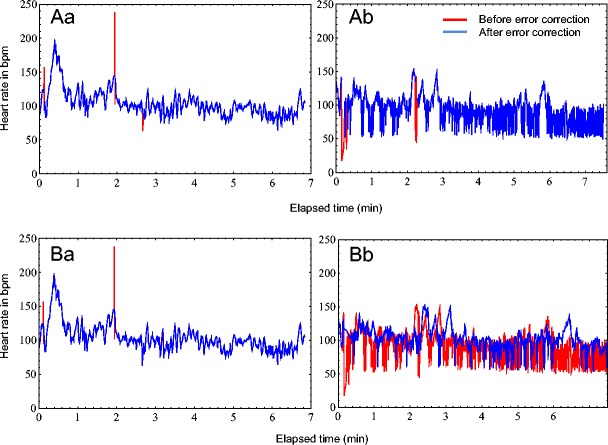
Heart rate (HR) both before (red) and after (blue) error correction using the AVEC method (Aa and Ab) and using the Polar error correction (Ba and Bb) for two different dogs. (**A**) Using the AVEC method: **a.** Dog 9 showed 0.56 % identified errors within the HR data; **b.** Dog 13 showed 0.75 % identified errors within the HR data. (**B**) With the Polar error correction method: **a.** 0.8 % errors were identified for the HR data of Dog 9; **b.** 20.5 % errors were identified for the HR data of Dog 13

When the Polar error correction method was applied to Threat 2, only the HR strings of two dogs were below the 5 % error limit; the strings of seven other dogs showed below 20 % errors; and all others were above 20 % errors. Polar identified significantly more errors than did AVEC (Wilcoxon signed-rank test: *n* = 24, *Z* = –4.286, *p* < .001; see Table 1). Furthermore, the standard deviation was higher for the uncorrected data than after either the AVEC or the Polar correction, but the standard deviation was higher after AVEC than after the Polar correction (Fig. 2Bb). For HRV parameters, we found that the SDNN, RMSSD, and pNN50 were higher in the uncorrected data than after both error correction methods. But all HRV parameters were significantly higher following the AVEC procedure than after error correction by the Polar tool. The HR means, as well as the medians, did not differ significantly between the uncorrected and corrected data for both error correction approaches. We also found no difference between the mean and median values between the AVEC and Polar methods (for statistics, see Table 1, for raw data see Appendix Table 4).

**Table 1:** Comparison of heart rate (HR) and heart rate variability (HRV) data for the Threat 2 situation, before and after both the AVEC and Polar error correction

HR Parameters	After Polar Compared With After AVEC	Before Correction Compared With After Polar	Before Correction Compared With After AVEC
Percentage of errors (%)	***p*** **< .001**	—	—
	*Z* = –4.286	—	—
Standard deviation (bpm)	***p*** **< .001**	***p*** **< .001**	***p*** **< .001**
	*t* = 4.945	*Z* = –4.114	*Z* = –4.086
Mean (bpm)	*p = .*621	*p = .*605	*p = .*548
	*t* = 0.501	*t* = 0.524	*t* = 0.610
Median (bpm)	*p = .*522	*p = .*513	*p = .*417
	*Z* = –0.641	*Z* = –0.654	*t* = –8.827
SDNN (ms)	***p*** **< .001**	***p*** **< .001**	***p*** **< .001**
	*Z* = –4.086	*Z* = –4.200	*Z* = –3.986
RMSSD (ms)	***p*** **< .001**	***p*** **< .001**	***p*** **< .001**
	*Z* = –3.786	*Z* = –4.286	*Z* = –4.229
pNN50 (%)	***p*** **< .001**	***p*** **< .001**	***p*** **< .001**
	*t* = 5.775	*t* = 6.119	*t* = 6.313

Student’s paired t test was used if the data were normally distributed, otherwise the Wilcoxon signed-rank test was used (*n* = 24 for all tests).

Significant results are given in bold.

Subsequently, Dogs 9 and 13 were taken as examples, to show how the HR data looked before and after the two different methods of error correction and to show how the different error correction approaches influenced the HR patterns (Fig. 2).

The dog HR patterns were characterized by high variability (Fig. 2). The data of Dog 13 showed not only a changed HR pattern after the Polar error correction, but also a time shift, which was probably caused by the Polar Pro Trainer 5 program not just replacing, but also inserting values. This may result in a different data length after the correction than before (Fig. 2Bb; Table 2).

**Table 2:**
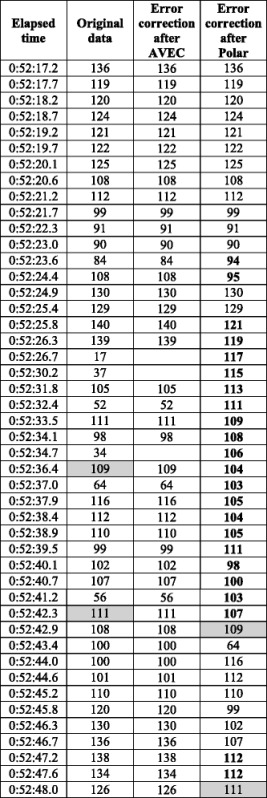
Heart rate (HR, in bpm) of Dog 13 for the first 30 s during the Threat 2 situation, before error correction, after AVEC, and after the Polar error correction method

Bold values were corrected and added by the Polar tool. Shaded values illustrate the time shift

### Effects of outlier treatment

Neither deleting outliers nor replacing them by means influenced the original data much (Table 3). The HR means only showed a difference of up to 0.21 % when comparing deleting or replacing values; the HR medians showed almost no change; and the HR standard deviations changed up to 0.87 %. However, the HRV parameters differed, depending on how the outliers were treated: The SDNN changed more when the error values were replaced by the mean (in four cases, over 1.3 %) than when they were deleted (less than 1 % difference). The RMSSD and pNN50 showed similar patterns. In the case of the RMSSD, the replacement of errors with means resulted in changes higher than 1.19 % in all cases. When error values were deleted, the data sets changed less than 1.05 %. The pNN50 changed by approximately 2 % when replaced by means, whereas after deleting values, the change was always below 2 %. This exploratory comparison confirms the sensitivity of the HRV parameters to even minor changes in the data string.

**Table 3:** Percentages of changes in heart rate (HR) and heart rate variability (HRV) parameters of a single dog, when approximately 1 %, 2 %, 3 %, 4 %, and 5 % of the values in the data set were randomly deleted or replaced by a mean

HR and HRV Parameters	% of Values Deleted					% of Values Replaced by Mean				
	0.94	1.87	2.81	3.98	4.92	0.94	1.87	2.81	3.98	4.92
HR Mean	0.21	0	0.10	0.10	–0.21	0	–0.10	0	0	–0.10
HR Median	1.15	0	0	0	0	0	0	0	0	0
HR Standard deviation	0	0	0.29	0.58	0.29	–0.29	–0.58	–0.58	–0.58	–0.87
SDNN	–0.71	–0.92	–0.59	–0.25	–0.21	–0.88	–1.38	–1.43	–1.43	–1.64
RMSSD	–1.04	–0.79	–0.35	0.20	0.65	–1.19	–1.49	–1.39	–1.24	–1.24
pNN50	0.17	0.86	1.90	1.55	1.72	0.34	1.55	2.41	2.41	2.24

## Discussion

We showed that our newly developed algorithm-supported visual error correction (AVEC) for HR data in dogs was mainly preferable over the standard Polar error correction tool because it decreases the likelihood of committing Type I errors, in two ways: first, AVEC results in significantly lower error rates, and thereby allows more data strings to be kept in the analysis, and second, it distorts the data variability significantly less, particularly with regard to HRV. In our present data set, the HR data of only two dogs would have been below the 5 % error rate with the Polar correction method, whereas with AVEC all 24 data sets remained in the analysis. Our results agree with conclusions from the literature indicating that HRV parameters especially are sensitive to only a few errors (Berntson & Stowell, 1998; Mulder, 1992), whereas the mean or median are less sensitive (Schendera, 2007).

With AVEC, each value is examined discretely by including only the neighboring values into the comparison, which makes this method flexible enough to apply even to data sets with high variation. Due to the peculiarities of dog HR variability, an automatic algorithm may wrongly delete values as outliers, which are part of the dog HR pattern, which was the case when using the Polar system tool for the error correction. This was also true for the error correction program of Kaufmann, Sütterlin, Schulz, and Vögele (2011), who processed part of our data with their program. Their algorithm (Berntson et al., 1990) wrongly identified HR values as outliers that were actually part of dog HR patterns (Suetterlin, personal communication), resulting in a percentage of errors similarly high to what we found from the Polar correction.

In general, it seems to be a necessity to visually inspect the data string and remove evident outliers (Schendera, 2007). This should be done even before applying a formal mathematical procedure, especially if outliers exceed a certain frequency (Berntson et al., 1990). When the error frequency is high, precautions should be taken when replacing them by a mean value, because the surrounding values may also be errors themselves. In contrast, particularly with HRV, deleting values may not only influence further analysis toward more conservative results, but may also shift it qualitatively, mainly because it can disturb the basic time series that is required for HRV analysis (Berntson & Stowell, 1998). In our data set, deleting outliers and replacing them by means did not influence the original data much, but HRV parameters were more sensitive to replacing errors by means than to simply deleting without replacement.

The limitations of AVEC include that it must still be performed by an experienced person and is relatively time-consuming, and hence only practicable for relatively short strings (minutes rather than hours). Also, the researcher has to make the final decision regarding whether or not a value is an outlier. However, we consider this a strength rather than a weakness. Also, behavioral data, if they are available, can be taken in account to support a decision. For example, the researcher may wish to check whether extreme HR episodes are linked to specific behaviors or environmental events.

Although our method was specifically developed for dog HR, it may also be appropriate for other species. For example, we have successfully applied it to humans and wolves, but the difference from the customized Polar error correction may be less than in the case of dogs.

We conclude that systems such as Polar are an easy-to-use alternative to conventional ECG systems, which are expensive and mainly designed for lab settings (von Borell et al., 2007), and therefore are hard to apply in unrestrained situations, as well as having evident constraints when working with private domestic dogs. However, to keep the likelihood of Type I errors within an acceptable range, error correction deserves proper attention.

## Appendix

**Table 4:** Comparison of heart rate and heart rate variability raw data for the Threat 2 situation, before and after the AVEC and the Polar error corrections

Dog	% Errors AVEC		Standard Deviation			Mean			Median			SDNN			RMSSD			pNN50		
	After AVEC	After Polar	Before Correction	After AVEC	After Polar	Before Correction	After AVEC	After Polar	Before Correction	After AVEC	After Polar	Before Correction	After AVEC	After Polar	Before Correction	After AVEC	After Polar	Before Correction	After AVEC	After Polar
1	1.39	4.30	22.03	20.71	19.87	118.32	117.96	118.00	115.00	115.00	116.00	92.10	85.70	84.10	59.00	26.50	24.60	8.10	6.40	6.20
2	1.25	37.00	27.45	27.30	21.85	107.06	107.06	110.00	106.00	106.00	109.00	179.60	179.00	116.50	170.60	164.30	63.30	53.20	52.00	23.60
3	1.65	28.40	30.91	31.04	31.18	85.50	85.84	85.99	87.00	87.00	89.00	354.30	356.70	359.10	308.40	306.90	174.40	59.10	58.20	40.90
4	0.53	45.80	27.80	27.26	13.99	84.86	85.00	88.00	85.00	85.00	88.00	282.20	277.40	125.20	330.70	321.80	100.20	63.70	63.30	29.40
5	1.29	9.80	22.48	19.47	19.97	89.37	88.00	89.00	87.00	87.00	87.00	151.20	146.50	147.10	107.30	101.70	88.60	45.00	44.80	39.70
6	0.84	50.00	37.37	37.46	17.35	93.20	92.99	72.00	86.00	86.00	75.00	297.70	298.10	226.10	289.20	289.80	178.80	60.40	60.80	49.10
7	1.26	33.80	42.31	41.72	20.71	115.40	115.21	103.00	106.00	106.00	99.00	208.20	202.10	132.50	194.20	176.30	102.90	42.80	41.70	29.60
8	0.15	29.30	43.06	43.06	28.25	108.24	108.00	99.00	99.00	99.00	97.00	249.30	249.40	206.90	185.00	184.90	122.50	49.30	49.20	41.30
9	0.56	0.80	22.62	22.00	22.10	107.49	107.31	107.39	102.00	102.00	102.00	101.80	100.20	101.20	44.70	38.90	40.80	15.80	15.20	15.00
10	0.23	39.50	18.99	18.71	18.85	124.22	124.51	136.00	122.00	123.00	134.00	77.50	68.50	62.50	53.70	21.20	12.80	3.20	2.90	0.90
11	1.61	55.60	35.44	34.81	36.59	111.79	112.08	127.64	107.00	107.00	120.00	194.70	190.10	139.50	132.90	118.70	40.40	36.90	35.50	9.10
12	0.54	13.00	34.60	34.01	28.86	109.42	109.33	106.02	102.00	102.00	102.50	192.60	181.40	173.00	121.20	85.10	90.10	31.80	31.40	28.90
13	0.75	20.50	23.31	22.80	17.86	95.59	96.04	101.12	98.00	98.00	101.00	234.70	199.00	135.10	311.60	262.80	132.50	55.70	55.20	31.90
14	4.52	34.10	88.91	37.17	23.47	119.06	119.04	116.44	112.50	113.00	113.00	545.90	173.00	121.40	508.30	152.30	63.30	42.40	39.70	16.90
15	0.93	58.70	35.37	32.82	24.42	142.00	142.38	162.12	133.00	134.00	168.00	188.60	86.20	63.80	180.60	34.80	10.50	10.90	9.90	0.80
16	1.51	12.10	26.65	25.49	19.72	80.65	80.38	77.89	72.00	72.00	72.00	218.30	207.70	182.90	158.90	131.00	100.00	45.30	44.10	41.30
17	1.38	22.00	20.13	20.02	16.76	95.56	95.22	92.50	90.00	90.00	88.00	131.70	131.30	115.10	107.70	106.20	90.20	56.40	56.10	49.10
18	0.93	12.30	17.47	17.10	16.01	98.48	98.26	98.12	98.00	98.00	98.00	114.40	113.10	104.70	129.40	124.90	108.80	58.10	57.50	49.10
19	0.58	15.10	33.11	32.68	14.68	102.70	102.72	94.52	95.00	95.00	94.00	156.30	145.60	105.90	128.70	96.50	74.80	36.70	35.90	33.50
20	0.87	19.00	20.24	19.64	15.63	85.61	86.09	86.85	86.00	86.00	86.00	226.20	189.90	141.40	269.20	109.40	139.10	62.90	62.20	47.60
21	1.29	44.70	37.32	36.24	25.37	77.87	77.55	68.92	67.00	67.00	62.00	409.70	386.40	366.90	433.60	419.60	352.50	76.30	75.80	55.70
22	0.95	11.80	20.54	19.84	17.96	90.27	90.82	90.70	88.00	88.00	88.00	195.20	153.80	140.80	218.50	150.00	131.10	49.40	48.40	42.50
23	1.84	26.30	32.76	31.21	25.64	111.96	111.08	110.67	106.00	105.00	108.00	168.60	157.00	131.60	140.70	115.80	69.90	33.60	31.80	21.70
24	1.03	22.20	33.14	32.60	18.21	114.87	115.27	104.91	108.00	108.00	104.00	152.10	139.50	108.30	119.00	83.90	80.80	39.90	38.80	36.80
